# Pigmented eccrine poroma in an atypical location^☆^

**DOI:** 10.1016/j.abd.2021.10.006

**Published:** 2022-07-07

**Authors:** Melissa de Almeida Corrêa Alfredo, Mariana Righetto de Ré Lai, Luciane Donida Bartoli Miot, Gabriela Roncada Haddad, Aline Lutz Garcia, Hélio Amante Miot

**Affiliations:** a Department of Dermatology, Faculty of Medicine, Universidade Estadual Paulista, Botucatu, SP, Brazil; b Department of Pathology, Faculty of Medicine, Universidade Estadual Paulista, Botucatu, SP, Brazil

**Keywords:** Poroma, Neoplasms, Skin neoplasms

## Abstract

Eccrine poroma is the term that includes benign neoplasms of the terminal duct of the eccrine sweat glands, which may clinically and dermoscopically resemble other melanoma and non-melanoma skin tumors. They are often located on the extremities (especially palms and soles), presenting as normochromic or erythematous papules and nodules, measuring up to 2 cm. Pigmented variants are uncommon, accounting for less than 20% of cases. This report describes a 37-year-old man who developed a large pigmented eccrine poroma on his right shoulder, causing diagnostic difficulty. Histopathological examination revealed a nodular neoplasm consisting of small, monomorphic, cuboidal cells, with ample, eosinophilic cytoplasm and well-defined borders, in addition to conspicuous intercellular bridges, with melanin deposits diffusely distributed inside them. The absence of cytological atypia, cellular pleomorphism, increased mitotic activity, and necrosis foci corroborated the diagnostic exclusion of porocarcinoma, which can develop from eccrine poroma.

Eccrine poromas (EP) are benign neoplasms of the sweat glands consisting of epithelial cells with distal tubular differentiation (acrosyringium), representing approximately 10% of all sweat gland tumors. They are often located on the acral regions, especially the palms and soles, due to the high density of eccrine glands in these areas but can affect any region of the skin.^1^ Clinically, EPs present as normochromic or erythematous papules and nodules, but rarely as vegetative and ulcerative tumors. Pigmented variants are uncommon and may mimic other pigmented tumors, such as melanoma and pigmented basal cell carcinoma.^2^

This report describes a young adult patient with an atypical, pigmented EP lesion on the shoulder, clinically resembling other pigmented neoplasms, emphasizing the relevance of histopathological examination for diagnostic confirmation.

A 37-year-old dark-skinned male reported that he had a tumor showing progressive growth on the posterior region of the right shoulder, for one year and a half (Fig. 1A). Clinical examination disclosed a brownish vegetative tumor with a crusted surface and an infiltrated erythematous base, measuring 4 × 3 cm. Dermoscopy of the lesion showed a homogeneous image, without no structures suggestive of basal cell carcinoma or any criteria for melanocytic lesions.

**Figure 1:**
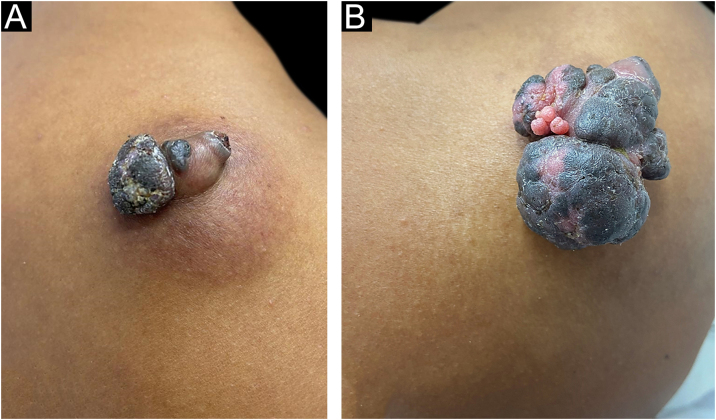
Pigmented eccrine poroma on the right shoulder. (A), Brownish tumor with infiltrated base, lobulated blackened areas, and crusted surface, at the first visit. (B), One year later, increase in tumor volume (5 × 4 cm), with a blackened, multilobulated appearance, and evident vegetative and exulcerated areas.

Due to the pandemic (COVID-19), the surgical excision was delayed for one year, leading to lesion growth. Upon reassessment, the patient had a vegetative, erythematous-violaceous tumor with a blackish-brown, infiltrated surface, with a slightly erythematous base, measuring 5 × 4 cm (Fig. 1B), which gave off a foul odor. The patient had no palpable lymph node enlargement or other relevant alterations on physical examination.

The lesion was submitted to total excision, with a safety margin, under the hypotheses of nodular melanoma, proliferating trichilemmal cyst, pigmented basal cell carcinoma, and pigmented dermatofibrosarcoma protuberans (Bednar's tumor).

The histopathological examination revealed a well-defined nodular neoplasm, connected with the epidermis, consisting of small, monomorphic, cuboidal cells, with ample, eosinophilic cytoplasm and well-defined borders, in addition to conspicuous intercellular Bridges. There were melanin deposits diffusely distributed inside the neoplasm. Moreover, intracytoplasmic lumens and ductular formations delineated by cuticles (Figs. 2‒Figure 3, Figure 4 ) were observed in some tumor cuboidal cells. The tumor lobules did not show stromal retraction or peripheral nuclear palisading.

**Figure 2:**
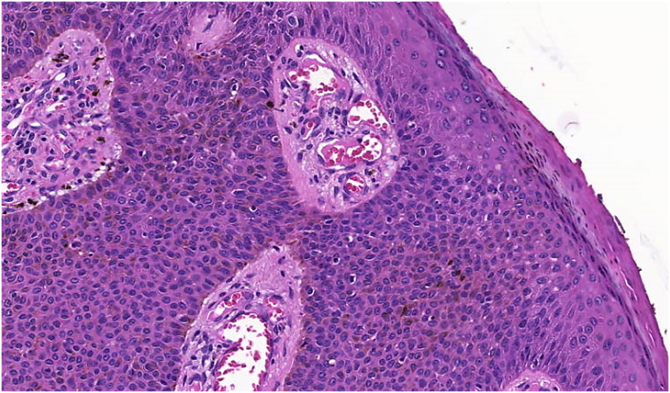
Pigmented eccrine poroma (Hematoxylin & eosin, ×100). Neoplasm consisting of small cuboidal cells with ample cytoplasm (smaller than the keratinocytes), with melanin deposition diffusely distributed within the neoplasm. The neoplasm is connected to the epidermis but there is no peripheral palisading.

**Figure 3:**
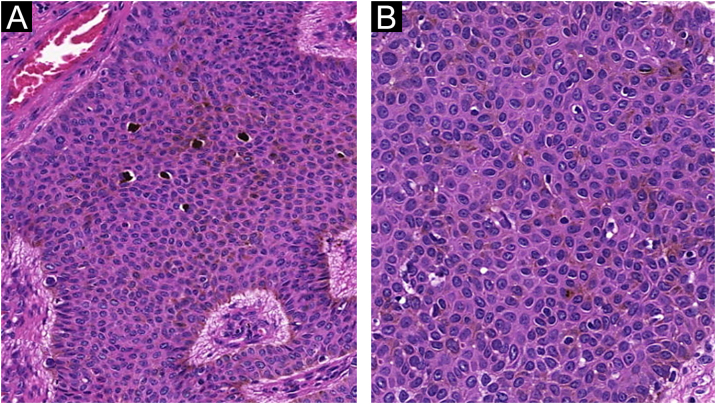
Pigmented eccrine poroma. (A), Area with evident intratumoral melanocytes (Hematoxylin & eosin, ×100). (B), Detail of the formation of incipient intracytoplasmic lumens inside the tumor (Hematoxylin & eosin, ×400).

**Figure 4:**
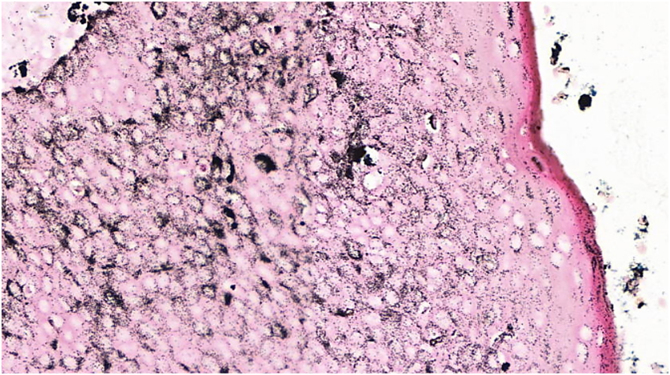
Pigmented eccrine poroma (Fontana-Masson, ×100). Diffuse intratumoral deposition of melanin granules, showing a greater intensity than that of the overlying epidermis.

The absence of cytological atypia, cellular pleomorphism, increased mitotic activity, and foci of necrosis corroborated the exclusion of porocarcinoma as a diagnostic possibility. Polyclonal CEA (carcinoembryonic antigen) immunostaining was negative (Fig. 5).

**Figure 5:**
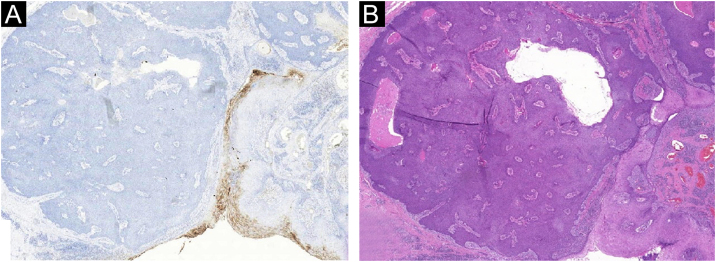
Pigmented eccrine poroma. (A), Negative immunostaining for polyclonal carcinoembryonic antigen (CEA). (B), Comparative image of the neoplasm (Hematoxylin & eosin, ×10).

The first EP description was reported by Pinkus et al. (1956), who described five cases of benign plantar tumors, whose histological assessment indicated a neoplasm that affected the terminal duct of the sweat glands.^3^

Histopathologically, EP is a benign tumor that originates in the intraepidermal portion of the sweat duct. Its etiology remains uncertain and it is a slow-growing tumor, with heterogeneous features; however, it is associated with trauma, radiation, and growth over scar tissue.^4^ The acral occurrence implies a differential diagnosis, mainly with myrmecia, squamous cell carcinoma (epithelioma *cuniculatum*), pyogenic granuloma and amelanotic nodular melanoma. Surgical excision with complete removal of the neoplasm is the treatment of choice, which leads to complete healing of the lesion.

The pigmented variant of EP is uncommon, corresponding to only 17% of cases, and has been previously described in individuals with different phototypes. Its diagnosis is challenging due to its rarity, and because it can be mistaken for other pigmented tumors such as melanoma, seborrheic keratosis, pigmented basal cell carcinoma, and Bednar's tumor.^5^, ^6^ These characteristics make histopathological analysis crucial for diagnostic confirmation and exclusion of malignancy. In these cases, there are numerous dendritic melanocytes dispersed among the tumor cells, giving the neoplasm the pigmented appearance. However, pigmented forms do not imply a different prognosis or local behavior from non-pigmented forms of EP.

Eccrine porocarcinoma, which can also be pigmented, is the main differential diagnosis on histopathological examination, inasmuch as it has a slow growth, and incisional biopsies may reveal components with apparent benign characteristics, delaying the diagnosis.^7^, ^8^ The malignant transformation of eccrine poromas is suggested by these intratumoral findings of porocarcinomas, which indicates the removal of the entire suspicious lesion, whenever possible, for the anatomopathological study with an extensive evaluation of the neoplasm, regarding cell atypia, circumscribed architecture, mitotic activity, foci of necrosis, and cell pleomorphism.^9^, ^10^ Immunohistochemical demonstration of positivity with polyclonal CEA in the cytoplasm of tumor cells and in glandular lumen formations, helps in the differentiation from other epithelial neoplasms. It should be noted that negative CEA immunostaining can occur in up to 23% of porocarcinomas, especially if the monoclonal antibody is used.^5^, ^11^

Dermatologists should be aware of the diagnosis of slow-growing pigmented tumors, even outside the palms and soles, due to the possibility of a pigmented EP, a tumor with a favorable prognosis after extensive surgical excision.

## Financial support

None declared.

## Authors' contributions

Melissa de Almeida Corrêa Alfredo: Design and planning of the study; approval of the final version of the manuscript; critical review of the literature; critical review of the manuscript; intellectual participation in the propaedeutic and/or therapeutic conduct of the studied cases.

Mariana Righetto de Ré Lai: Approval of the final version of the manuscript; critical review of the literature; critical review of the manuscript; intellectual participation in the propaedeutic and/or therapeutic conduct of the studied cases.

Luciane Donida Bartoli Miot: Design and planning of the study; approval of the final version of the manuscript; critical review of the manuscript; intellectual participation in the propaedeutic and/or therapeutic conduct of the studied cases.

Gabriela Roncada Haddad: Approval of the final version of the manuscript; critical review of the manuscript; intellectual participation in the propaedeutic and/or therapeutic conduct of the studied cases.

Aline Lutz Garcia: Approval of the final version of the manuscript; critical review of the manuscript; intellectual participation in the propaedeutic and/or therapeutic conduct of the studied cases.

Hélio Amante Miot: Approval of the final version of the manuscript; critical review of the manuscript.

## Conflicts of interest

None declared.
